# Salivary gland dysfunction and salivary redox imbalance in patients with Alzheimer’s disease

**DOI:** 10.1038/s41598-021-03456-9

**Published:** 2021-12-13

**Authors:** Anna Zalewska, Anna Klimiuk, Sara Zięba, Olga Wnorowska, Małgorzata Rusak, Napoleon Waszkiewicz, Izabela Szarmach, Krzysztof Dzierżanowski, Mateusz Maciejczyk

**Affiliations:** 1 Department of Conservative Dentistry, Medical University of Bialystok, Sklodowskiej St. 24A, 15-276 Bialystok, Poland; 2 Independent Dentistry Laboratory, Medical University of Bialystok, Sklodowskiej St. 24A, 15-276 Bialystok, Poland; 3 Doctoral Studies, Medical University of Bialystok, Sklodowskiej St. 24A, 15-276 Bialystok, Poland; 4 Psychogeriatric Ward of SPP ZOZ (Independent, Public Mental Health Care Centre) in Choroszcz, Brodowicza St. 1, 16-070 Choroszcz, Poland; 5 Department of Hematological Diagnostics, Medical University of Bialystok, Waszyngtona St. 15 a, 15-276 Bialystok, Poland; 6 Department of Psychiatry, Medical University of Bialystok, Brodowicza St. 1, 16-070 Choroszcz, Poland; 7 Department of Orthodontics, Medical University of Bialystok, Waszyngtona St. 15 a, 15-276 Bialystok, Poland; 8 Department of Hygiene, Epidemiology and Ergonomics, Medical University of Bialystok, Mickiewicza St. 2c, 15-022 Bialystok, Poland

**Keywords:** Biochemistry, Biomarkers, Diseases

## Abstract

Alzheimer’s disease (AD) is associated with the deposition of β-amyloid in the brain. AD accounts for over 50% of cases of dementia which results from disturbances in redox homeostasis. Indeed, increased intensity of protein oxidation and nitration as well as lipid peroxidation is observed in brain areas with considerable amounts of amyloid plaques and neurofibrillary tangles. However, little is known about the oxidoreductive balance of salivary glands in AD patients. Therefore, the aim of this study was to evaluate the antioxidant barrier and oxidative/nitrosative stress biomarkers in stimulated saliva and blood of AD patients. The study was participated by 25 AD patients and 25 non-demented controls without neurological diseases or cognitive impairment, matched by age and gender to the study group. The number of patients was determined based on a previous pilot study (test power = 0.9). We found a significant decrease in the activity of erythrocyte superoxide dismutase (SOD) and glutathione peroxidase (GPx), increased activity of catalase (CAT) and reduced concentration of plasma non-enzymatic antioxidants (uric acid, UA and reduced glutathione, GSH). In contrast, in the stimulated saliva of AD patients we observed significantly decreased activity of all antioxidant enzymes (SOD, CAT and GPx) as well as concentration of GSH compared to the control group. The content of lipid (malondialdehyde, MDA) and protein (advanced oxidation protein products, AOPP; advanced glycation end-products, AGE) oxidation products as well as biomarkers of nitrosative stress (peroxynitrite, nitrotyrosine) was significantly higher in both saliva and plasma of AD patients compared to the controls. In AD patients, we also observed a considerable decrease in stimulated saliva secretion and salivary total protein content, and an increase in salivary β-amyloid concentration. In conclusion, AD results in redox imbalance towards oxidative reactions, both at the level of the oral cavity and the entire body. General redox balance disturbances do not coincide with salivary redox balance disturbances. Reduction in stimulated saliva secretion in AD patients reflects secretory dysfunction of the parotid glands.

## Introduction

Neurodegenerative diseases are characterized by progressive degeneration of cells of the central and peripheral nervous system, which consequently leads to impairment of cognitive and motor functions. One of neurodegenerative diseases is Alzheimer’s disease (AD) which, according to the available data, accounts for approximately 80% of all dementia cases^1^. AD is usually preceded by a period of mild cognitive impairment (MCI), i.e., short-term memory impairment, speech loss, being ‘lost for words’ as well as impaired orientation, concentration and attention. In the advanced stage of the disease, which usually increases with the patient’s age, we can observe symptoms of depression, apathy, sleep disorders, delusions and hallucinations. For a long time, β-amyloid (Aβ) deposits and neurofibrillary tangles were considered the main causative factors in the development of AD. Nowadays, it is known that the pathogenesis of this disease is more complex, and neuroinflammation and oxidative stress (OS) play a significant role in it. The latter phenomenon is described as imbalance between excessive formation of oxygen (ROS) and nitrogen (RNS) free radicals and their neutralization. Disruption of this delicate oxidoreductive balance results in oxidative modification/destruction of numerous cellular components, leading to cell/organ dysfunction and development of diseases. The brain is highly susceptible to redox imbalance due to its high-energy demand and high oxygen consumption in mitochondria, which makes them the main target of oxidative damage. However, it is also significant that Aβ can build into the lipid bilayer of brain cells and enhance ROS production, and other important factors are the abundance of peroxidation-prone polyunsaturated fatty acids, high iron concentrations and a relatively poor panel of antioxidants in brain cells^2^.

Interestingly, numerous scientific publications indicate that patients with AD have systemic manifestations accompanying nervous system dysfunction, which suggests that the disease affects both the brain and the peripheral organs, including the salivary glands^3,4^.

In the salivary glands, not only has the presence of Aβ deposits been confirmed, but also the expression of Aβ and TAU protein in their acinar epithelial cells^3,4^. A significant increase in the salivary Aβ_1-42_ in patients with mild and moderate AD was also observed compared to patients with very advanced stage of the disease^3^, and significantly elevated t-TAU/p-TAU ratio was found in AD patients^4^. The results of Shi et al.^4^ suggest that salivary TAU can take a phosphorylated form which is essential for the development and progression of AD. In contrast to the results of Bermejo-Pareja et al.^3^, Kim et al.^5^ observed positive correlation between salivary Aβ and the stage of AD progression.

Redox imbalance in the salivary glands and saliva of AD patients has not really been evaluated. However, it has been demonstrated that OS is a key factor in hyposalivation (reduced salivary secretion) in a group of patients with dementia of different etiology and varying degree of progression. Regardless of the type and severity of dementia (without confirmed AD), reduced antioxidant response of the salivary glands and increased modification of salivary cell elements were observed. It was noteworthy that reduced glutathione (GSH) deficiency is a factor differentiating patients with severe dementia from those with mild and moderate type of dementia^6,7^.

The aim of the present study was to find out how Alzheimer’s disease affects the functioning of the salivary glands and oral structures, as well as the salivary redox equilibrium parameters. We also looked for links between salivary redox biomarkers and the criteria for diagnosis, duration and severity of AD.

## Materials and methods

### Patients

The research was approved by the Bioethics Committee in Białystok, No. APK.002.95.2020. Patients qualified for the experiment had had the aim and methodology of the study explained. Informed consent was obtained from all participants. 10 patients with AD did not agree to have saliva and blood samples collected and they did not participate in the study. The study was performed in accordance with the Declaration of Helsinki and relevant local guidelines.

25 AD (15 Polish women, 10 Polish men, Caucasian) patients under observation in the Psychogeriatric Ward of SPP ZOZ (Independent Public Mental Health Care Centre) in Choroszcz were qualified for the study. The study also included 25 non-demented controls (15 Polish women, 10 Polish men, Caucasian) without neurological diseases or cognitive impairment, attending dental check-ups at the Department of Conservative Dentistry of the MUB (Medical University of Bialystok). The control group was matched to the study group in terms of gender and age. All AD patients were diagnosed by an experienced professor of psychiatry (N. W.) and a neuropsychologist (O. W.) according to the NINCDS-ADRDA criteria of 2007^8^. Establishing the diagnosis required finding cognitive decline (via a clinical mental examination) as well as impairment of social or occupational functions. The Mini-Mental State Examination (MMSE) was used to assess cognitive functions^9^. All patients in the study and control group had biochemical blood tests (vitamin B12, folic acid, thyroid function assessment, vitamin D3) performed. Patients in the study group also had brain imaging tests (brain MRI) done. MRI was routinely performed in the hospital to confirm the diagnosis of AD. Detailed MRI results are confidential and therefore cannot be used in the manuscript. However, 100% confirmation of the disease is possible only after the death of the patient.

Exclusion criteria: history of hemorrhagic or ischemic stroke, traumas within the cerebral part of the skull, severe depression not allowing to establish the diagnosis of given dementia or determine its level, vascular dementia (based on DSM-III-R criteria / the FLAIR sequence of MRI confirming abnormalities in the medial temporal lobes, typical of inflammation or focal lesion of vascular origin), tumors within the brain, insulin resistance, diabetes, thyroid diseases, obesity, psoriasis, Parkinson’s disease, Huntington’s disease, deficiency of vitamin B12 or folic acid. Patients in the study and control groups did not abuse alcohol or smoke cigarettes. They had not taken antibiotics or supplements during the 6 months preceding material collection and had not changed their diet (AD patients – information obtained mostly from interviews with their family or hospital staff).

## Research material

The material for the study was collected between 6 and 7 a.m. between October and February, upon fasting and before oral hygiene procedures of the patients.

Due to the fact that it was impossible to collect unstimulated saliva (no saliva in the oral cavity), only stimulated whole saliva was used for the study. Saliva collection procedure was initiated in an environment familiar to the patient, usually at the bedside, after drinking a glass of water and a 5-min conversation. Saliva was collected by an experienced and trained dentist (S. Z.). Patients had 100 µL of citric acid sprayed on the tip of the tongue every 30 s for 10 min, and the saliva collected in the mouth was taken with a pipette (the procedure of spitting into a tube was not feasible in every case). The collected saliva was set aside in a container with ice and then centrifuged within 30 min from its collection (5000 g, 20 min, + 4 °C; MPW 351, MPW Med. Instruments, Warsaw, Poland). Salivary supernatant fluid enriched with butylated hydroxytoluene (BHT, 5 µL 0.5 M BHT in acetonitrile per 0.5 mL of salivary supernatant) was retained for the study and stored at − 84 °C for no longer than 6 months. The addition of BHT prevents oxidation of the sample^10^.

A dental examination was then performed by two experienced and previously calibrated dentists (A. K., A. Z). Objective symptoms of dry mouth^11^ and DMFT index (D—decayed, M—missing, F—filled, T—teeth) were assessed. If teeth were present, the gingival index (GI) was assessed as well^12^. Not all patients consented to the measurement of pocket depth; therefore, we refrained from this measurement.

Venous blood (10 mL) was collected by qualified hospital staff. Blood was collected using the S-Monovette® K3 EDTA blood collection system (Sarstedt, Nümbrecht, Germany). Within 30 min after collection, blood samples were centrifuged (1500 g, 10 min, + 4 °C; MPW 351, MPW Med. Instruments, Warsaw, Poland). None of the samples underwent hemolysis. After separation of plasma, erythrocytes were rinsed three times with 0.9% cold NaCl solution and hemolyzed. BHT (5 µL 0.5 M BHT in acetonitrile per 0.5 mL of sample) was added to the plasma and blood cell lysate. The samples were stored at ~ 84 °C for no longer than 6 months.

### Materials and chemicals

2,2′-azino-bis(3-ethylbenzothiazoline-6-sulfonic acid) diammonium (Cat. No. 10102946001), 2-mercaptoethanol (Cat. No. M6250), 5,5’-dithiobis(2-nitrobenzoic acid) (Cat. No. D218200), acetic acid (Cat. No. 33209-M), adenochrome (Cat. No. A5752), chloramine T (Cat. No. 402869), diethylenetriaminepentaacetic acid (Cat. No. 17969), dihydronicotinamide adenine dinucleotide phosphate tetrasodium (Cat. No. NADPH-RO), epinephrine hydrochloride (Cat. No. E4642), ethylenediaminetetraacetic acid (Cat. No. ED), ferrous ion (Cat. No. 450278), flavin adenine dinucleotide disodium (Cat. No. F8384), glycerol (Cat. No. G5516), hydrochloric acid (Cat. No. H1758), hydrogen peroxide (Cat. No. H1009), lactate dehydrogenase (Cat. No.427211), N-(1-naphthyl)-ethylenediamine dihydrochloride (Cat. No. N9125), nitrate reductase (Cat. No. 10981249001), o-dianisidine (Cat. No. D9143), phenol (Cat. No. 33517), phosphate buffer (Cat. No. P5244), phosphoric acid (Cat. No. 466123), potassium iodide (Cat. No. 207969), potassium persulfate (Cat. No. 216224), potassium phosphate monobasic (Cat. No. P5379), potassium thiocyanate (Cat. No. P3011), pyruvic acid (Cat. No. 107360), sodium acetate (Cat. No. 241245), sodium carbonate (Cat. No. S2127), sodium chloride (Cat. No. S9888), sodium hydroxide (Cat. No. 06203), sodium phosphate (Cat. No. 342483), sodium phosphate dibasic (Cat. No. 94046), sulfanilamide (Cat. No. 33626), sulfuric acid (Cat. No. 339741), thioflavin T (Cat. No. T3516), xylenol orange (Cat. No. 398187) was provided by Sigma Aldrich (St. Louis, MO, USA). In turn, commercial kit to UA determination was provided by QuantiChrom™ (Cat. No. DIUA-250, Harward, CA, USA). Nitrotyrosine ELISA commercial kit was provided by Immundiagnostik AG (Cat. No. K 7829, Bensheim, Germany). IL-1 beta Human ELISA commercial Kit was purchased from R&D Systems (Cat. No. DLB50, Canada, Minneapolis, MN, USA). Human lactoferrin ELISA kit was obtained from EIAab (Cat. No. E-EL-H5200, Wuhan, China).

### Antioxidant assays

Superoxide dismutase (SOD, E.C. 1.15.1.1) activity was determined by measuring the rate of inhibition of adrenaline oxidation to adrenochrome using the spectrophotometric method at a wavelength of 480 nm. It was assumed that one unit of SOD activity inhibits the oxidation of adrenaline by 50%^13^.

The activity of catalase (CAT, E.C. 1.11.1.6) was measured spectrophotometrically at 240 nm wavelength based on the rate of hydrogen peroxide decomposition. One unit of CAT activity was defined as the amount of the enzyme that decomposes 1 mmol of hydrogen peroxide per one minute^14^.

Salivary peroxidase (Px, E.C. 1.11.1.7) activity was determined spectrophotometrically at 412 nm by measuring the decrease in the absorption of 5,5-dithio-bis-(2-nitrobenzoic acid) (DTNB) to thionitrobenzoic acid. Measurements were made five times at intervals of 30 s. Erythrocyte glutathione peroxidase (GPx, EC 1.11.1.9) activity was assayed spectrophotometrically at 340 nm wavelength by determining the reduction of organic peroxides in the presence of NADPH. It was assumed that one unit of GPx catalyzes the process of oxidation of 1 μmol NADPH per one minute^15^.

Uric acid (UA) level was measured by the colorimetric method, using the commercial kit (QuantiChrom™ Uric Acid DIUA-250; BioAssay Systems, Harward, CA, USA), as instructed by the manufacturer. In this method, 2,4,6-tripyridyl-s-triazine forms a blue complex with Fe^3+^ and UA. The absorbance was measured at 630 nm wavelength.

The concentration of reduced glutathione (GSH) was measured by the colorimetric method developed by Ellman with the use of DTNB. The absorbance was measured at 412 nm^16^.

### Redox status

Total antioxidant capacity (TAC) was determined by the colorimetric method, which is based on the measurement of the ability to neutralize the radical cation ABTS^+^ [2,2-azino-bis- (3-ethylbenzothiazoline-6-sulfonate)] under the influence of antioxidants contained in the tested samples. The change in absorbance of the solution was measured at a wavelength of 660 nm^17^.

Total oxidation status (TOS) was assessed colorimetrically. This method is based on the oxidation of iron (2^+^) ions to iron (3^+^) ions in the presence of oxidants contained in the sample, followed by detection of Fe^3+^ ions by xylene orange. TOS was calculated from the standard curve for hydrogen peroxide and is reported in nM of H_2_O_2_ equivalent/mg of total proteins. TOS determination was performed in three samples^18^.

The oxidative stress index (OSI) was calculated according to the formula: OSI = TOS/TAS × 100^19^.

### Oxidative damage products

Protein advanced glycation end products (AGE) concentration was estimated spectrofluorimetrically by measuring AGE-specific fluorescence at 350 nm/440 nm wavelength. For AGE determination in plasma, samples were diluted 1:50 (v/v) in phosphate-buffered saline (PBS)^20^.

AOPP content was analyzed spectrophotometrically at 340 nm wavelength by measuring the oxidative capacity of iodine ion. For AOPP measured in plasma, samples were diluted 1:50 (v/v) in phosphate-buffered saline (PBS)^21^.

The concentration of malondialdehyde (MDA) was measured against blank sample as thiobarbituric acid reactive substances (TBARS), using a reference curve for 1,1,3,3-tetramethoxypropane.

### Nitrosative stress

Nitric oxide (NO) level was measured by the colorimetric method using sulfanilamide and N-(1-naphthyl)-ethylenediamine dihydrochloride. The absorbance of the obtained product was measured at 490 nm wavelength^22,23^.

Peroxynitrite level was measured colorimetrically based on peroxynitrite-mediated nitration leading to the formation of nitrophenol. The absorbance of the obtained complex was measured at 320 nm wavelength^24,25^.

Nitrotyrosine level was measured using the ELISA commercial kit (Nitrotyrosine ELISA; Immundiagnostik AG, Bensheim, Germany) according to the manufacturer’s instructions.

### Amyloid cross β-structure (Aβ)

To evaluate the β-amyloid formation, 90 μL of the analyzed samples was placed on the microplate and 10 μL of Thioflavin T was added. The intensity of the amyloid associated with thioflavin T fluorescence was measured at 385/485 nm wavelength^26^.

### Interleukin- 1 Beta (IL-1β)

The concentration of interleukin 1β (IL-β) was determined with the use of commercial ELISA kit (IL-1 beta Human ELISA Kit, R&D Systems, Canada, Minneapolis, MN, USA) based on double-antibody sandwich method according to the manufacturer's instruction.

### Lactoferrin (LF)

ELISA method was used to determine the salivary lactoferrin concentration according to the manufacturer's instruction (Human lactoferrin ELISA kit, EIAab, Wuhan, China).

### Statistical data analysis

Statistical analysis was performed using GraphPad Prism 8 for macOS (GraphPad Software, La Jolla, CA, USA). Normality of distribution was determined using the Kolmogorov–Smirnov test. Student's t-test or U-Mann Whitney test were used for group comparisons. Results were presented as median (minimum—maximum) and mean ± SD. The assumed statistical significance was *p* < 0.05. The number of patients was set a priori based on our previous study. The level of significance was set at 0.05, and the power of the study was 0.9. The diagnostic usefulness of salivary and plasma biomarkers was examined using ROC (Receiver Operating Curve) analysis by determining the area under the curve (AUC). The sensitivity and specificity and the cut-off point, which was characterized by the highest sensitivity and specificity, were given.

## Results

Although the severity of AD is different in males and females, we did not find any significant gender differences in the parameters studied. Perhaps it was caused by the small size of both subgroups of patients. Therefore, the results are presented for men and women combined.

### Clinical and dental characteristics

There were no significant differences in biochemical parameters or blood morphology between patients with Alzheimer’s disease and the control group. The flow of stimulated saliva as well as total protein concentration in SWS were significantly lower (91%↓ *p* ≤ 0.0001, 42%↓ *p* = 0.0022) in patients with AD compared to the controls. Clinical and dental data of Alzheimer’s disease patients and the control group are presented in Table 1.

**Table 1:** Stomatological and clinical characteristics of AD patients and the control group.

	C		AD		*P*-value
	Median (minimum–maximum)	Mean ± SD	Median (minimum–maximum)	Mean ± SD	
Sex (Men/Female)	10/15		10/15		NS
Age (years)	83.6 (66–90)	82.1 ± 6.67	82.5 (66–90)	81.19 ± 6.765	NS
Time from diagnosing the disease (years)	–	–	9 (5–13)	9.043 ± 2.30569	–
**Stomatological characteristics**					
SWS total protein (μg/mL)	2314 (1379–3872)	2368 ± 647.2	1338 (203.8–4391)	1597 ± 1065	0.0022
SWS flow (mL/min)	1.465 (0.5–2)	1.364 ± 0.479	0.12 (0.09–0.26)	0.09 ± 0.06325	< 0.0001
DMFT	28.44 (25.44–29.98)	29.45 ± 0.56	28.66 (27.34–29.98)	29.98 ± 0.88	NS
GI	1.58 (1.34–2.3)	1.58 ± 0.07	1.99 (1.59–2.18)	2.05 ± 0.15	NS
**Blood count and biochemical characteristics**					
Leukocytes (10^3^/µl)	7.87 (3.55–9.7)	6.41 ± 2.211	6.3 (3.9–12.6)	6.72 ± 2.114	NS
Erythrocytes (10^6^/µl)	4.21 (3.92–5.61)	4.185 ± 0.563	4.075 (3.33–6.1)	4.233 ± 0.6395	NS
HGB (g/dL)	13.23 (11.9–17.42)	12.89 ± 1.123	12.95 (9.7–14.2)	12.74 ± 1.081	NS
HCT (%)	39.32 (33.24–50.02)	37.96 ± 2.806	38.05 (29.3–42.3)	37.87 ± 2.902	NS
Glucose (mg/dL)	79 (70–94)	115.6 ± 46.64	78 (68–96)	116.9 ± 46.22	NS
CRP (mg/L)	3.2 (0.2–12.45)	23.8 ± 75.17	3.4 (0.1–37.1)	23.4 ± 77.19	NS
AST (U/L)	19 (10–46)	25.89 ± 12.42	21 (12–63)	26.26 ± 11.48	NS
ALT (U/L)	14 (7–55)	17.98 ± 8.67	17 (7–35)	18.42 ± 8.329	NS
Na^+^ (mmol/L)	143.6 (136.5–140.2)	142.2 ± 3.321	142.3 (135.9–145.1)	141.5 ± 2.922	NS
K^+^ (mmol/L)	4.58 (3.87–5.8)	9.87 ± 30.25	4.215 (3.71–147)	10.81 ± 30.42	NS
TSH (µIU/mL)	1.4 (1.45–4.69)	1.925 ± 1.102	1.34 (0.69–4.73)	1.857 ± 1.201	NS
Vit. D_3_ (ng/mL)	20 (12.13–53.23)	18.67 ± 13.46	16.19 (3–43.67)	17.37 ± 12.32	NS
Vit. B_12_ (pg/mL)	314.7 (123–542)	256.6 ± 132.5	214.3 (50–456.3)	222.7 ± 121.2	NS
Folic acid (ng/mL)	2.34 (1.23–16.55)	3.105 ± 3.021	2.28 (0.88–12.53)	3.324 ± 3.028	NS
**Cognitive function**					
MMSE	28 (26–29)	16 ± 5.43	15 (0–20)	14 ± 4.69	NS
**Concomitant diseases**					
Hypertension n (%)	14 (56)		16 (64)		NS
Diabetes n (%)	2 (8)		3 (12)		NS
CHD n (%)	5 (20)		6 (24)		NS
Atherosclerosis n (%)	3 (12)		3 (12)		NS
Osteoporosis n (%)	1 (4)		2 (8)		NS
**Drugs**					
Donepezil n (%)	0		12 (48)		< 0.0001
Rivastigmine n (%)	0		10 (40)		< 0.0001
Galantamine n (%)	0		3 (12)		< 0.0001
ACEi n (%)	12 (48)		11 (44)		NS
β-blockers n (%)	5 (20)		6 (24)		NS
CCB n (%)	8 (32)		7 (28)		NS
Loop diuretics n (%)	4 (16)		4 (16)		NS
Metformin (%)	2 (8)		3 (12)		NS
Alendronate (%)	1 (4)		1 (4)		NS

AD—Alzheimer’s disease; C—control group; SWS—stimulated whole saliva; DMFT—decayed, missing, filled teeth index; GI—gingival index; HGB—hemoglobin; HCT—hematocrit; CRP—C-reactive protein; AST—aspartate transaminase; ALT—alanine transferase; Na^+^—sodium; K^+^—potassium; TSH—thyroid stimulating hormone; Vit. D_3_—vitamin D_3_; Vit. B_12_—vitamin B12; MMSE—Mini Mental State Examination; CHD—coronary heart disease; ACEi—angiotensin converting enzyme inhibitors; CCB—calcium channel blockers.

**Figure 1:**
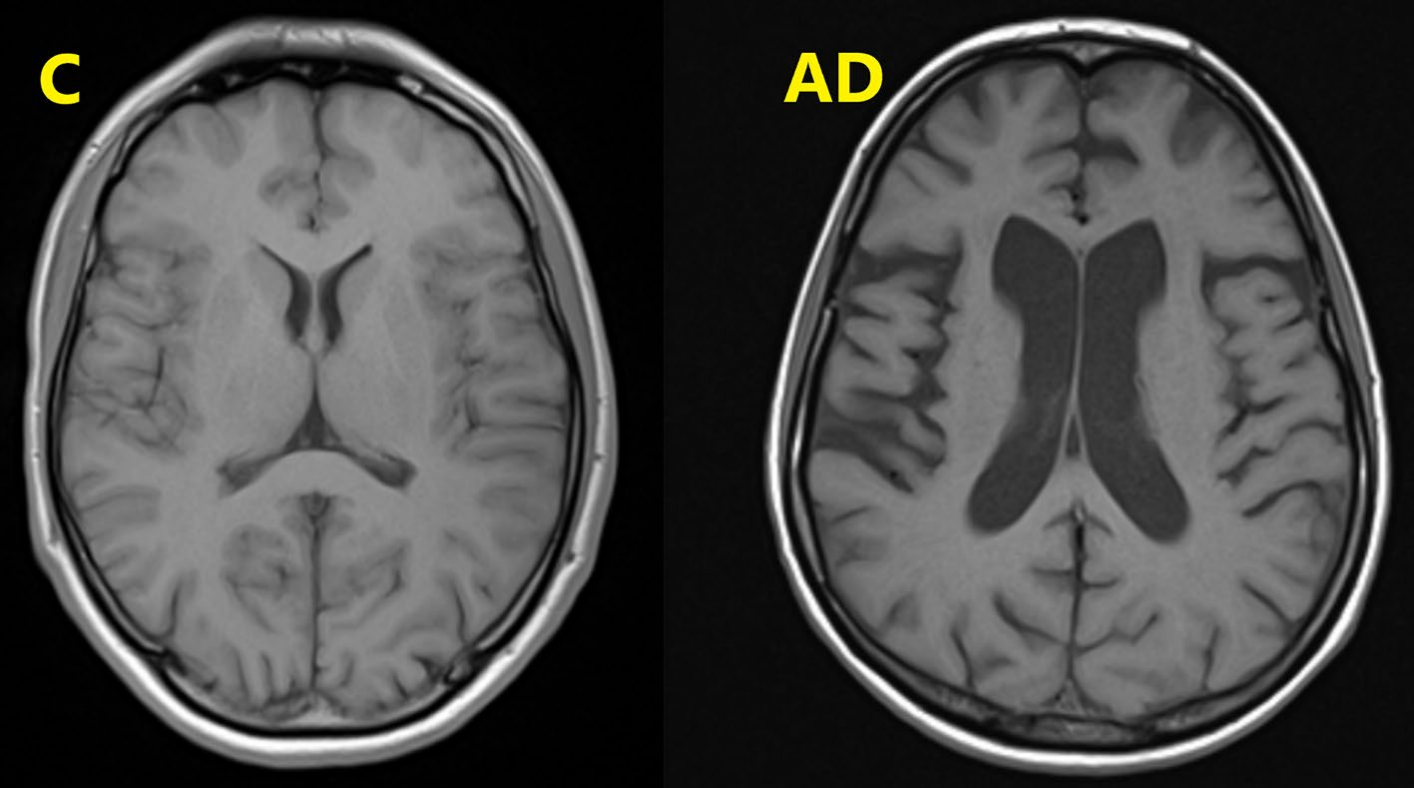
Visual examples of the brain atrophy changes in patient suffering from Alzheimer's disease (AD; atrophy of the temporal and frontal lobes) with ventricular enlargement, in comparison to the non-demented control person (C).

Visual examples of the brain atrophy changes in patient suffering from Alzheimer's disease (AD; atrophy of the temporal and frontal lobes) with ventricular enlargement, in comparison to the non-demented control person (C) are presented in Fig. 1.

In the AD group, significantly poorer hydration of the vermilion zone and buccal mucosa was observed. The condition of tongue mucosa was also worse compared to the control group (Table 2).

**Table 2:** Assessment of dryness of the oral cavity in AD patients and healthy controls.

	Assessment n (%)	AD	C	*p*
Vermilion zone hydration	0	0	3 (12%)	< 0.0001
	1	1 (4%)	18 (72%)	
	2	2 (8%)	2 (8%)	
	3	22 (88%)	2 (8%)	
Buccal mucosa hydration	1	0	15 (60%)	< 0.0001
	2	0	10 (40%)	
	3	25 (100%)	0	
Condition of tongue mucosa	1	0	23 (92%)	< 0.0001
	2	25 (100%)	2 (8%)	
Palpation of salivary glands	1	1 (4%)	25 (100%)	< 0.0001
	2	24 (96%)	0	

AD—Alzheimer’s disease; C—control group.

### Redox homeostasis

**Figure 2:**
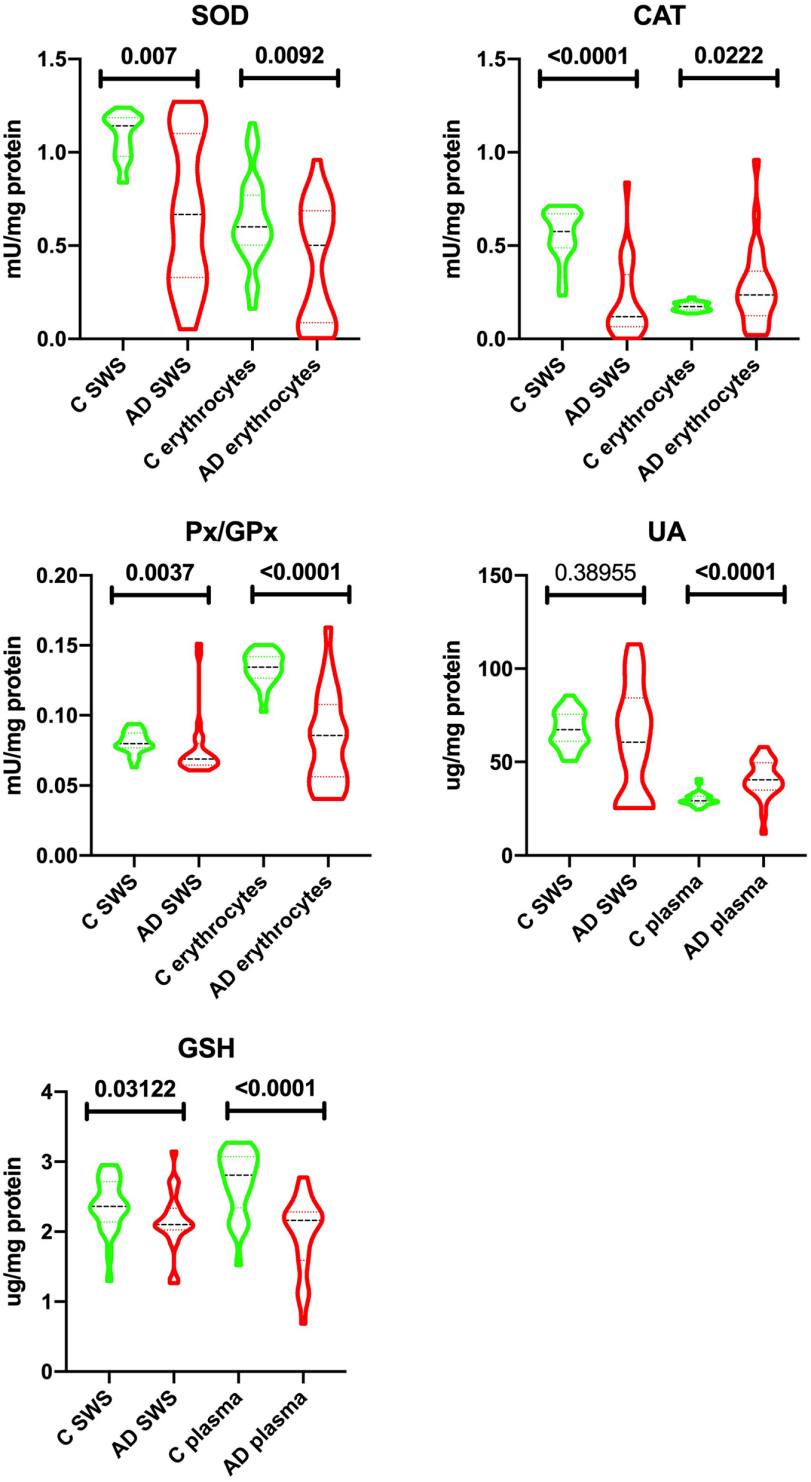
Salivary and erythrocytes/plasma antioxidant barrier in patients with Alzheimer’s disease and the control group. SOD—superoxide dismutase; CAT—catalase; Px—peroxidase; GPx—glutathione peroxidase; UA—uric acid; GSH—glutathione; C—control group; AD—Alzheimer’s disease group; SWS stimulated whole saliva.

**Figure 3:**
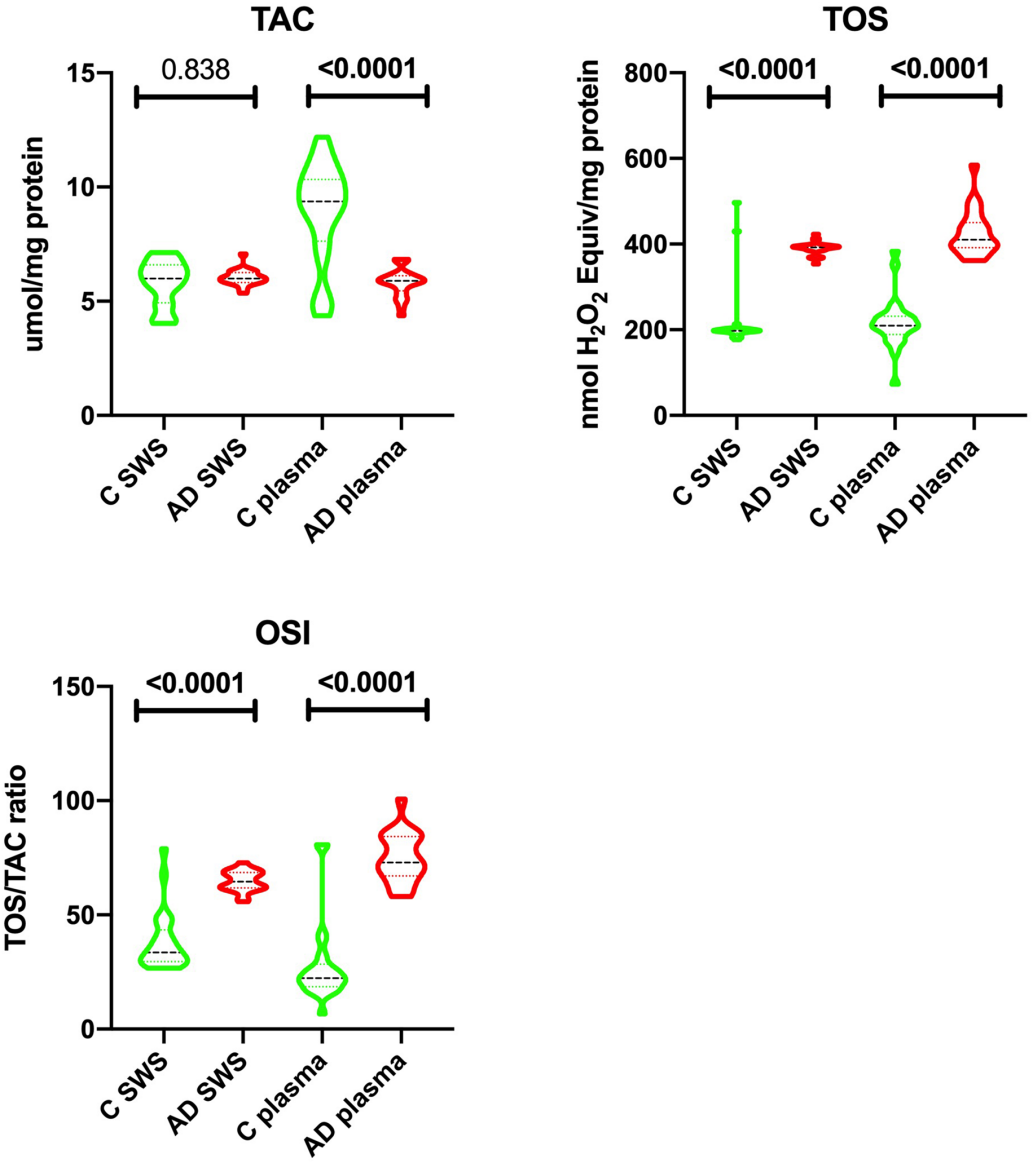
Salivary and erythrocytes/plasma redox status in patients with Alzheimer’s disease and the control group. TAC—mean total antioxidant capacity; TOS—mean total oxidant status; OSI—oxidative stress index; C—control group; AD—Alzheimer’s disease group; SWS—stimulated whole saliva.

**Figure 4:**
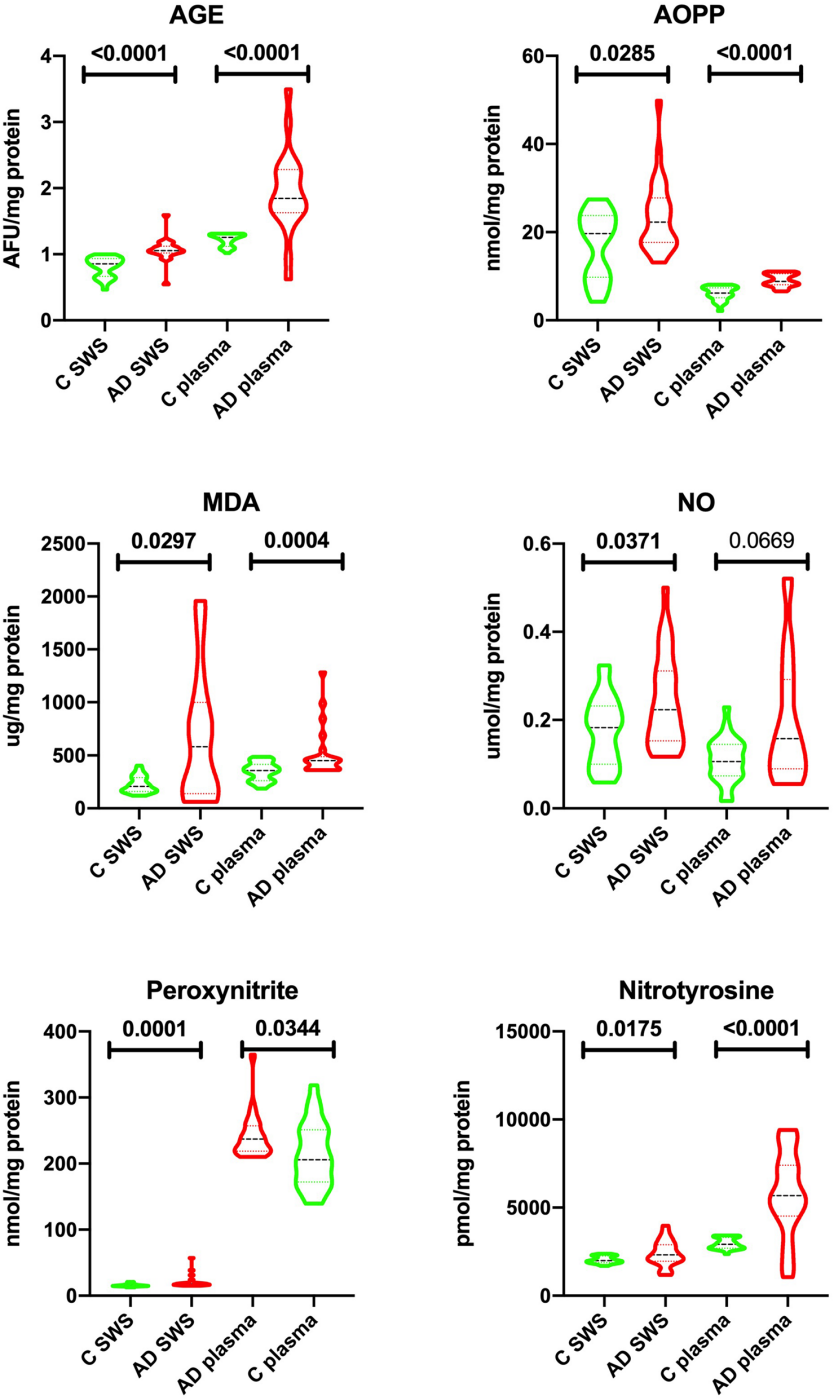
Salivary and erythrocytes/plasma oxidative and nitrosative stress in patients with Alzheimer’s disease and the control group. AGE—advanced glycation end products; AOPP—advanced oxidation protein products; MDA—malondialdehyde; NO—nitric oxide; C—control group; AD—Alzheimer’s disease group; SWS—stimulated whole saliva.

The results of redox balance parameters in saliva, erythrocytes and plasma of AD patients and controls are presented in Figs. 2, 3 and 4.

### Antioxidants

The activity of SOD, CAT and GPx in SWS of AD patients was significantly lower compared to the control group (↓42% *p* = 0.0007, ↓79% *p* ≤ 0.0001, ↓15% *p* = 0.0037, respectively). Similarly, the concentration of GSH in the SWS of patients with Alzheimer’s disease was considerably lower (↓11% *p* = 0.0312) compared to the control group. The concentration of UA in the SWS of AD patients as compared to the control group was at a similar level, not reaching statistical significance.

On the other hand, significantly lower SOD and GPx activity and GSH levels (↓17% *p* = 0.0092, ↓36% *p* ≤ 0.0001, ↓23% *p* ≤ 0.0001, respectively) were observed in erythrocytes/plasma of the participants from the study group. CAT erythrocyte activity and concentration of plasma UA were significantly higher (↑27% *p* = 0.0222, ↑28% *p* ≤ 0.0001, respectively) in the group of patients with Alzheimer’s disease compared to the control group (Fig. 2).

## Redox status

In SWS of patients with Alzheimer’s disease, TOS as well as OSI were considerably higher (↑50% *p* ≤ 0.0001, 48% *p* ≤ 0.0001, respectively) than in the control group. At the same time, there was no significant difference in the level of salivary TAC between those two groups. The plasma of patients with Alzheimer’s disease was characterized by a significantly lower level of TAC (↓37% *p* ≤ 0.0001) with a considerably higher levels of TOS and OSI (↑49% *p* ≤ 0.0001, ↑69% *p* ≤ 0.0001) compared to the control group (Fig. 3).

### Oxidative damage products

The group of patients with Alzheimer’s disease was characterized by a considerably higher concentrations of AGE, AOPP and MDA (↑19% *p* ≤ 0.0001, ↑12% *p* = 0.0285, ↑64% *p* = 0.0297, respectively) in SWS compared to the control group. Similar results were observed in the plasma of the study group, where the concentrations of AGE, AOPP, MDA were also considerably higher (↑32% *p* ≤ 0.0001, ↑30% *p* ≤ 0.0001, ↑21% *p* = 0.0004, respectively) compared to the controls (Fig. 4).

### Nitrosative stress

The concentrations of NO, peroxynitrite and nitrotyrosine in SWS of AD patients were significantly higher than in the control group (↑18% *p* = 0.0371, ↑13% *p* = 0.0001, ↑14% *p* = 0.0175, respectively). Similar results were obtained in the plasma of patients from the study group where the concentrations of NO, peroxynitrite and nitrotyrosine were considerably higher than in the control group (↑33% *p* = 0.0669, ↑5% *p* = 0.0344, ↑48% *p* ≤ 0.0001, respectively) (Fig. 4).

### Others biomarkers

The concentration of Aβ in SWS of patients with Alzheimer’s disease was significantly higher (↑30% *p* ≤ 0.0001) compared to the control group. In the plasma, there was no significant difference in Aβ concentration between those two groups.

The group of patients with Alzheimer’s disease was characterized by a considerably lower concentrations of LF (↑16% *p* = 0.0211) in SWS compared to the control group.

In SWS and plasma of patients with Alzheimer’s disease, the IL-1β—concentration was significantly higher (↑24% *p* ≤ 0.0001, ↑59% *p* = 0.0092) compared to the control group (Table 3).

**Table 3:** The concentration of salivary and erythrocytes/plasma Aβ, LF, IL-1β and IL-1β in patients with Alzheimer’s disease and the control group.

Biomarker	Saliva					Erythrocytes/Plasma				
	C		AD		*P*-value	C		AD		*P*-value
	Median (minimum–maximum)	25–75% Percentile	Median (minimum–maximum)	25–75% Percentile		Median (minimum–maximum)	25–75% Percentile	Median (minimum–maximum)	25–75% Percentile	
Aβ (AFU/mg protein)	5049 (678.7–6512)	3691–5476	7236 (5402–18,396)	6351–9299	< 0.0001	70,487 (26,787–85,117)	56,906–74,762	70,948 (33,935–102,063)	60,802–78,992	0.5405
LF (μg/mg protein)	29.97 (12.32–42.57)	23.81–35.36	24.52 (9.866–45.34)	21.17–45.34	0.0211	–				
IL-1β (ng/mg protein)	70.85 (46.24–96.09)	63.21–75.44	88.47 (55.21–155.3)	78.29–101.4	< 0.0001	25.6 (13.39–37.09)	20.32–30.28	44.26 (24.32–73.17)	39.5–46.29	0.0092

C—control group; AD—Alzheimer’s disease; Aβ—amyloid beta, LF—lactoferrin; IL-1β—Interleukin 1 Beta.

### Correlations

Correlations between salivary redox biomarkers and activity of the salivary glands as well as AD severity are presented in Table 4. It is particularly noteworthy that we found a statistically significant positive correlation between SWS flow and MMSE and a negative correlation between SWS flow and AOPP in the SWS of AD patients. Within the scope of assays including salivary antioxidants in the SWS of patients with AD, we observed positive correlations between Px as well as SOD and time elapsed from AD diagnosis, and Aβ level and SOD activity. Additionally, the concentration of AOPP in SWS of the study group correlated positively with buccal mucosa hydration and negatively with GSH (Table 5).

**Table 4:** Receiver operating characteristic (ROC) analysis of salivary redox biomarkers of patients with Alzheimer’s disease and the control group.

Biomarker	Saliva							
	AUC	95%Cl	*P*-value	Cut off	Sensitivity%	95%Cl	Specificity%	95%Cl
**Antioxidant barrier**								
SOD (mU/mg protein)	0.7774	0.6292 to 0.9256	0.001	< 1.028	69.57	49.13 to 84.40%	68.00	48.41 to 82.79%
CAT (nmol H_2_O_2_/min/mg protein)	0.9183	0.8267 to 1.000	< 0.0001	< 0.4197	82.61	62.86 to 93.02%	84.00	65.35 to 93.60%
GPx (mU/mg protein)	0.7409	0.5841 to 0.8976	0.0043	< 0.07697	73.91	53.53 to 87.45%	72.00	52.42 to 85.72%
UA (μg/mg protein)	0.5739	0.3950 to 0.7528	0.3804	< 66.40	52.17	32.96 to 70.76%	52.00	33.50 to 69.97%
GSH (ng/mg protein)	0.6836	0.5258 to 0.8415	0.0313	< 2.276	72.73	51.85 to 86.85%	72.00	52.42 to 85.72%
**Redox status**								
TAC (μmol/mg protein)	0.5183	0.3442 to 0.6923	0.8284	> 5.999	52.17	32.96 to 70.76%	52.00	33.50 to 69.97%
TOS (nmol/mg protein)	0.92	0.8137 to 1.000	< 0.0001	> 367.6	91.30	73.20 to 98.45%	92.00	75.03 to 98.58%
OSI (TOS/TAC ratio)	0.936	0.8473 to 1.000	< 0.0001	> 59.10	90.00	69.90 to 98.22%	92.00	75.03 to 98.58%
**Oxidative and nitrosative stress**								
AGE (AFU/mg protein)	0.9357	0.8505 to 1.000	< 0.0001	> 0.9610	86.96	67.87 to 95.46%	88.00	70.04 to 95.83%
AOPP (nmol/mg protein)	0.68	0.5307 to 0.8293	0.029	> 20.19	56.00	37.07 to 73.33%	56.00	37.07 to 73.33%
MDA(μg/mg protein)	0.6876	0.5075 to 0.8677	0.0298	> 243.7	66.67	45.37 to 82.81%	68.00	48.41 to 82.79%
NO (ng/mg protein)	0.672	0.5229 to 0.8211	0.037	> 0.1961	56.00	37.07 to 73.33%	56.00	37.07 to 73.33%
Peroxynitrite (nmol/mg protein)	0.8163	0.6927 to 0.9398	0.0002	> 16.74	63.64	42.95 to 80.27%	79.17	59.53 to 90.76%
Nitrotyrosine (pmol/mg protein)	0.7018	0.5405 to 0.8631	0.018	> 2107	63.64	42.95 to 80.27%	64.00	44.52 to 79.75%
**Others**								
Aβ (AFU/mg protein)	0.9491	0.8941 to 1.000	< 0.0001	> 5861	86.36	66.67 to 95.25%	84.00	65.35 to 93.60%
LF (μg/mg protein)	0.6896	0.5368 to 0.8424	0.0215	< 26.36	64.00	44.52 to 79.75%	64.00	44.52 to 79.75%
IL-1β (ng/mg protein)	0.8528	0.7420 to 0.9636	< 0.0001	> 76.95	84.00	65.35 to 93.60%	84.00	65.35 to 93.60%

AUC—area under curve; SOD—superoxide dismutase; CAT—catalase; GPx—glutathione peroxidase; UA—uric acid; GSH—glutathione; TAC—mean total antioxidant capacity; TOS—mean total oxidant status; OSI—oxidative stress index; AGE—advanced glycation end products; AOPP—advanced oxidation protein products; MDA—malondialdehyde; NO—nitric oxide, Aβ—amyloid beta, LF—lactoferrin; IL-1β—Interleukin 1 Beta.

**Table 5:** Receiver operating characteristic (ROC) analysis of redox biomarkers in erythrocytes/plasma of patients with Alzheimer’s disease and the control group.

Biomarker	Erythrocytes/Plasma							
	AUC	95%Cl	*P*-value	Cut off	Sensitivity%	95%Cl	Specificity%	95%Cl
**Antioxidant barrier**								
SOD (mU/mg protein)	0.684	0.5458 to 0.8222	0.0097	< 0.5543	60.00	40.74 to 76.60%	62.00	48.15 to 74.14%
CAT (nmol H_2_O_2_/min/mg protein)	0.69	0.5164 to 0.8636	0.0226	> 0.1822	68.00	48.41 to 82.79%	66.67	46.71 to 82.03%
GPx (mU/mg protein)	0.9167	0.8235 to 1.000	< 0.0001	< 0.1211	87.50	69.00 to 95.66%	88.00	70.04 to 95.83%
UA (μg/mg protein)	0.8617	0.7396 to 0.9837	< 0.0001	> 33.37	87.50	69.00 to 95.66%	88.00	70.04 to 95.83%
GSH (ng/mg protein)	0.8455	0.7330 to 0.9579	< 0.0001	< 2.293	77.27	56.56 to 89.88%	76.00	56.57 to 88.50%
**Redox status**								
TAC (μmol/mg protein)	0.855	0.7232 to 0.9868	< 0.0001	< 6.199	83.33	64.15 to 93.32%	84.00	65.35 to 93.60%
TOS (nmol/mg protein)	0.9927	0.9761 to 1.000	< 0.0001	> 363.1	95.45	78.20 to 99.77%	96.00	80.46 to 99.79%
OSI (TOS/TAC ratio)	0.9467	0.8731 to 1.000	< 0.0001	> 60.51	90.48	71.09 to 98.31%	92.00	75.03 to 98.58%
**Oxidative and nitrosative stress**								
AGE (AFU/mg protein)	0.9048	0.7792 to 1.000	< 0.0001	> 1.311	90.48	71.09 to 98.31%	92.00	75.03 to 98.58%
AOPP (nmol/mg protein)	0.9309	0.8598 to 1.000	< 0.0001	> 7.789	86.36	66.67 to 95.25%	84.00	65.35 to 93.60%
MDA (μg/mg protein)	0.7909	0.6631 to 0.9187	0.0006	> 393.4	68.18	47.32 to 83.64%	68.00	48.41 to 82.79%
**NO (ng/mg protein)**								
Peroxynitrite (nmol/mg protein)	0.6845	0.5210 to 0.8480	0.0344	> 224.4	57.14	36.55 to 75.53%	58.33	38.83 to 75.53%
Nitrotyrosine (pmol/mg protein)	0.8419	0.6929 to 0.9909	< 0.0001	> 3357	80.95	60.00 to 92.33%	80.00	60.87 to 91.14%
**Others**								
Aβ (AFU/mg protein)	0.5543	0.3840 to 0.7245	0.5297	> 70,825	61.90	40.88 to 79.25%	60.00	40.74 to 76.60%
IL-1β (ng/mg protein)	0.9456	0.8839 to 1.000	< 0.0001	> 34.07	84.00	65.35 to 93.60%	84.00	65.35 to 93.60%

AUC—area under curve; SOD—superoxide dismutase; CAT—catalase; GPx—glutathione peroxidase; UA—uric acid; GSH—glutathione; TAC—mean total antioxidant capacity; TOS—mean total oxidant status; OSI—oxidative stress index; AGE—advanced glycation end products; AOPP—advanced oxidation protein products; MDA—malondialdehyde; NO—nitric oxide, Aβ—amyloid beta; IL-1β—Interleukin 1 Beta.

### ROC analysis

**Figure 5:**
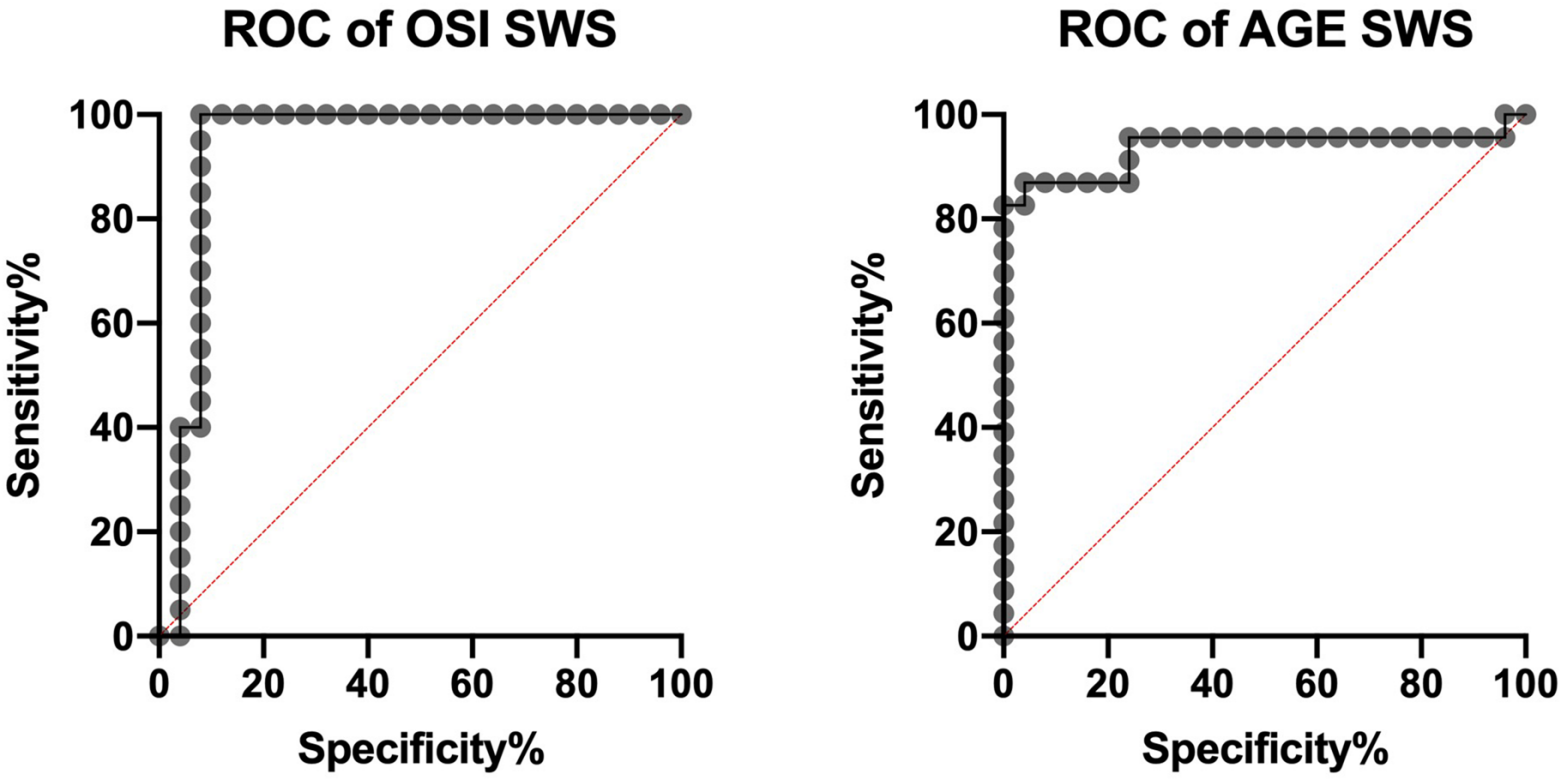
Receiver operating characteristic (ROC) analysis of OSI and AGE in stimulated whole saliva of patients with Alzheimer’s disease and the control group. AGE—advanced glycation end products; OSI—oxidative stress index; SWS—stimulated whole saliva.

The ROC analysis showed that most of the salivary redox biomarkers significantly differentiated group of patients with Alzheimer’s disease with the control group. The assessment of salivary CAT, GPx and GSH (sensitivity = 82.61%, specificity = 84.00%, *p* < 0.0001; sensitivity = 73.91%, specificity = 72.00%, *p* = 0.0043; sensitivity = 72.73%, specificity = 72.00%, *p* = 0.0313) clearly differentiates the AD patients from the control. Similarly, the salivary levels of OSI, AGE, Aβ and IL-1β (sensitivity = 90.00%, specificity = 92.00%, *p* < 0.0001; sensitivity = 86.96%, specificity = 88.00%, *p* < 0.0001; sensitivity = 86.36%, specificity = 84.00%, *p* < 0.0001; sensitivity = 84.00%, specificity = 84.00%, *p* < 0.0001) significantly distinguished the patients with AD from the control patients (Table 6, Fig. 5).

Interestingly, ROC analysis revealed a significantly high diagnostic value of GPx, UA and GSH assay in erythrocytes/plasma of patients with Alzheimer’s disease compared to the healthy controls (sensitivity = 87.50%, specificity = 88.00%, *p* < 0.0001; sensitivity = 87.50%, specificity = 88.00%, *p* < 0.0001; sensitivity = 77.27%, specificity = 76.0000%, *p* < 0.0001). Similarly, the plasma levels of TAC, TOS, OSI, AGE and AOPP (sensitivity = 83.33%, specificity = 84.00%, *p* < 0.0001; sensitivity = 95.45%, specificity = 96.00%, *p* < 0.0001; sensitivity = 90.48%, specificity = 92.00%, *p* < 0.0001; sensitivity = 90.48%, specificity = 92.00%, *p* < 0.0001; sensitivity = 86.36%, specificity = 84.00%, *p* < 0.0001) as well as nitrotyrosine and IL-1β (sensitivity = 80.95%, specificity = 80.00%, *p* < 0.0001; sensitivity = 84.00%, specificity = 84.00%, *p* < 0.0001) differentiating AD patients from the control group.

**Table 6:** Statistically significant correlations between redox biomarkers and clinical parameters.

Pair of variables	r	*p*
**AD**		
MMSE and SWS flow	0.908	< 0.0001
MMSE and plasma MDA	0.955	< 0.0001
SWS AOPP and SWS flow	− 0.672	0.002
SWS AOPP and SWS GSH	− 0.607	0.003
SWS Px and time elapsed from the diagnosis	0.877	< 0.0001
SWS SOD and time elapsed from the diagnosis	0.677	0.004
SWS SOD and SWS Aβ	0.732	0.0001
**C**		
AOPP and hydration of buccal mucosa	0.747	< 0.0001

AD—Alzheimer’s disease; MMSE—Mini-Mental State Examination; SWS—stimulated whole saliva; MDA—malondialdehyde; AOPP—advanced oxidation protein products; GSH—reduced glutathione; GPx—glutathione peroxidase; SOD—superoxide dismutase; Aβ—amyloid β; C—control group.

## Discussion

The main results of the presented study indicate that although salivary total antioxidant status (TAC) in AD patients was unaltered, we observed increased oxidative modification of cellular elements of the salivary glands and structures present in the oral cavity. Saliva is not only the secretion of the salivary glands, but it also contains exfoliated oral mucosal epithelium and gingival fluid reflecting the redox status of periodontal tissues. The finding that unstimulated salivary flow from the submandibular glands is impaired in AD patients has already been published^27^. However, it can be concluded that in the AD patients from our experiment the submandibular glands were completely dysfunctional. Despite using several methods of collecting unstimulated saliva, we were unable to collect the material. Even gentle attempts to collect saliva (using a pipette) from the mucous membranes of the lips, palate and cheeks failed. Moreover, we demonstrated high secretory failure of the parotid glands that secrete stimulated saliva. Despite providing hydration of the patient’s oral cavity (each patient drank a glass of water, and nurses reported that patients regularly drink various types of liquids) and collecting the material in autumn and winter (at room temperature of 20–21 °C), secretion of stimulated saliva in AD patients was reduced compared to their peers of the control group, as normal stimulated saliva secretion starts with the value of 0.7 mL/min. Stimulated secretion was at the level of 0.12 mL/min, which indicates severe hyposalivation. The cause of reduced salivary secretion in AD patients is unknown. Saliva secretion is initiated by reflex-induced nerve impulses. Controlling this process depends on neurotransmitters released at nerve ends in the salivary glands. Typical neurotransmitters associated with water secretion are acetylcholine (ACh) and numerous neuropeptide neurotransmitters^28^. In the brain of AD patients we observed increased activity of acetylcholinesterase, an enzyme which breaks down ACh, followed by decreased ACh levels^29^. This deficit in the cholinergic function was connected with loss of memory as well as cognitive and learning ability in AD individuals^29^. Sayer et al.^30^ reported decreased salivary concentration of ACh in AD patients who did not respond to AChE-I treatment. The positive correlation between SWS and Mini-Mental State Examination may prove that declined cholinergic conductivity could be a cause of hyposalivation. It should be noted that AD patients included in our experiment are treated with cholinesterase inhibitors (AChE-I) (Table 1). The reduction in SWS compared to the control and the lack of unstimulated secretion in each of the AD patients studied may suggest that these patients belong to the group of the so-called non-responders to AChE-I^30^. The negative correlation between AOPP levels and SWS may also be considered as some kind of explanation. It was demonstrated that protein oxidation can accelerate the formation of toxic protein aggregates in the nucleus and cytoplasm of the nerves^31^. This may inhibit neurotransmitter release or reduce salivary gland innervation and thus decrease secretory response of the salivary glands. Decreased protein levels in the SWS of patients with AD vs healthy controls may evidence reduced activity of the sympathetic nervous system, whose stimulation determines protein synthesis in the salivary glands. Due to the selection of the control group (age of patients), we excluded age-related salivary gland changes (fatty and fibrous degeneration) as a cause of salivary gland dysfunction. Many comorbidities and accompanying treatments may also affect saliva secretion and composition. These include comorbidities (hypertension, diabetes, coronary heart disease, atherosclerosis, osteoporosis) and medications taken by participants in our study. Nevertheless, the number of comorbidities and medications increases with age, so selecting patients with AD alone was not possible. It should be underlined that both patients and controls had similar general diseases and were taking similar groups of drugs, so connecting hyposalivation with a particular disease or drug-related condition would be an oversimplification. With such significant deficiency of saliva, it is not surprising to find poor hydration of the vermilion zone, buccal mucosa or tongue condition. The positive correlation between buccal mucosal hydration and AOPP concentration in the control group may result from increased oxidative modification of moisturizing proteins, i.e., mucins and other glycoproteins, accompanying the older age, which entails changes in the coating properties of saliva. Assessment of xerostomia (subjective perception of dryness in the oral cavity) was not possible in every case; therefore, we refrained from presentation of the results.

Analysis of the results obtained in plasma and red cell lysate revealed a significant decrease in SOD and GPx activity (↓17%, ↓39%, respectively), increase in CAT activity (↑17%), decrease in the concentration of non-enzymatic antioxidants (↓38% UA, ↓25% GSH, ↓39% TAC) and the existence of general OS (↑95% TOS, ↑227% OSI, ↑26% MDA, ↑47% AGE, ↑42% AOPP) and nitrosative stress (↑4% peroxynitrite, ↑94% nitrotyrosine). TAC is the resultant capacity of a given biological material to counteract specific oxidation reactions ^32^. It is believed that TAC level corresponds to the antioxidant capacity of non-enzymatic antioxidants in the sample. TOS reflects the level of all free radicals and non-free radical molecules. OSI is the so-called oxidative stress index, that is the ratio of antioxidants to oxidants present in the assayed material^33^.

Changes in the redox balance in SWS are shifted towards oxidative processes similarly to plasma/blood cells, although not exactly to the same extent. In stimulated saliva of AD patients, we observed significantly decreased activity of all antioxidant enzymes: SOD, CAT and GPx (↓42%, ↓80%, ↓15%, respectively) compared to the control group. Decreased parameters of these components of the antioxidant barrier may result mainly from highly increased production of ROS (98% ↑TOS) which consistently utilize antioxidants in their combating. It may also be due to oxidative modifications of protein chains of the mentioned enzymes (12% ↑AOPP, 20% ↑AGE), leading to their inactivation, as observed in the brain of AD patients^34^. Decreased GPx activity demonstrates antioxidant failure as well as impairment of other functions (not related to redox balance) of the parotid glands. As the only type of proteins studied, GPx is synthesized exclusively in the acinar cells of the salivary glands and is recognized as a marker of proper function of the parotid glands^35,36^. Interestingly, reduction of SOD and GPx activity in the SWS of AD patients correlated positively with the time elapsed from AD diagnosis; in addition, decreased SOD activity correlated positively with Aβ levels in the SWS. The latter correlation may be caused by high affinity of Aβ to bind Cu^2+^, which deprives saliva of this trace element^37^. Cu^2+^ is a cofactor of the dismutation reaction catalyzed by SOD^38^. Moreover, the ROC analysis showed that GPx significantly differentiated the group of patients with Alzheimer’s disease from the control group. Further studies are needed to confirm or rule out the hypothesis that decreased salivary SOD/GPx activity may be a specific biomarker of AD and help differentiate or assess the severity of the degree or duration of this disease, as is the case with lactoferrin. Reduced concentration of salivary lactoferrin is considered one of the indicators helpful in differentiating and diagnosing the early stages of AD. This salivary parameter is also used in predicting the development and progression of cognitive disorders in AD^39^. The LF concentration in SWS of our AD patients was also significantly lower than the control, but it did not show a significant correlation with the time elapsed since the diagnosis of the disease. It is worth recalling that labial salivary gland biopsy and saliva samples evaluation are diagnostic tools for AD diagnosis or prediction. Aβ is highly expressed in salivary epithelial cells and thus has high saliva concentration in patients with AD or at increased risk of developing AD^40^.

GSH deficiency (↓11%) accompanying negative correlation between GSH and AOPP may be due to increased oxidation of proteins that form oral cavity structures. Indeed, the main function of GSH is to maintain a reduced state of the thiol groups of proteins. The reduction of GSH may also result from deficiency of substrates involved in the regeneration of GSH. Another cause may be oxidative damage to GSH reductase, an enzyme that catalyzes the conversion of GSSG (oxidized form of glutathione) to GSH, with NADPH as the reducing cofactor^41^. A decrease in NADPH generation observed in the brain of AD patients^42^ could hinder the re-synthesis of salivary GSH. Reduction in GSH concentration in the saliva may enhance OH^.^ formation, thus increasing the ROS load and be the cause of the increase in the reported concentrations of peroxynitrite (↑15%) and nitrotyrosine (↑16%) in the SWS of AD patients. GSH is known as the more potent detoxification agent of peroxynitrite^41^. It was evidenced that peroxynitrite is involved in the pathogenesis of AD. It was observed that peroxynitrite simultaneously induces hyperphosphorylation, nitration and accumulation of tau protein^43^. The thus modified tau protein (p-tau) aggregates to form intracellular neurofibrillary tangles, exerting a toxic effect^43^. It is noteworthy that there was greater reduction in plasma GSH level (↓25%) than in the SWS level (↓11%) in AD patients, with simultaneous greater increase in plasma nitrosative stress expressed by nitrotyrosine levels (↑94% vs ↑16%).

Only UA and TAC in stimulated saliva of AD patients did not differ compared to the controls. Despite unchanged TAC levels, salivary antioxidant systems were unable to counterbalance the increased ROS/RNS formation (↑TOS) and prevent the development of OS (↑91% OSI, ↑AOPP, ↑AGE, ↑180% MDA). The reason for the accumulation of products of cellular elements oxidation/peroxidation could undoubtedly be malfunctioning of the repair systems responsible for the removal of defective macromolecules, which is typical in the course of AD^44^. Increased MDA concentration in SWS suggests the existence of advanced stages of free radical processes in the cells of salivary glands as well as oral cavity structures of AD patients compared to the control group^45^, as membrane lipid molecules are thought to undergo oxidative modification with generation of reactive aldehydes at higher concentrations of ROS than proteins^46^. Interestingly, we demonstrated a positive correlation between serum MDA concentration and MMSE. It can be assumed that the level of MDA reflects the degree of peroxidation of unsaturated fatty acids of the biological membranes and thus changes of membrane fluidity. Membrane fluidity determines the normal functioning of cells, including neurons, so an increase in serum MDA content could be linked to neuronal loss in AD.

Biomarkers are required to improve the diagnosis, monitoring, and progression of the diseases. In the case of AD diagnosis, the best-validated biomarkers are pTau and Aβ_42_; the sensitivity and specificity of which is about 90–95%^47,48^. Attempts were made to evaluate the usefulness of many plasma biomarkers, but they did not show sufficient sensitivity and specificity^49^. All of these biomarkers were also evaluated in saliva. However, the conclusions of the studies are ambiguous and generally do not support its high diagnostic utility^1,50^. In our study, the ROC analysis showed that OSI and AGE differentiated patients with Alzheimer's disease significantly from the control group. Of course, these are only preliminary studies that require confirmation on a larger number of patients.

The alterations in salivary redox balance are associated with chronic inflammation, which is one of the causes of AD. Salivary microRNAs can become potential biomarkers for the accurate and precise identification of inflammatory markers in AD patients. Unfortunately, the small amount of saliva collected from patients does not allow for a more detailed examination. We could only show a significant increase in IL-1β concentration and a reduction in LF concentration in SWS of AD patients compared to the control. For this reason, we were also unable to determine the recognized salivary biomarkers of AD, i.e. *α*-synuclein, Aβ1-42, Aβ1-40, Total-Tau, and Phospho-tau. This is the weak point of this publication. Furthermore, we were unable to correlate imaging studies of patients' brains with biochemical parameters in saliva. These data could allow us to conclude whether salivary redox balance biomarkers may be used in conjunction with MRI to diagnose AD patients accurately. Of course, we will undertake such research in the future.

## Conclusions

1. Alzheimer’s disease results in redox imbalance towards oxidation reactions, both at the level of the oral cavity and the entire body.
2. General redox balance disturbances do not coincide with salivary redox balance disturbances, which means that both processes occur independently.
3. Reduction in the activity of key antioxidant enzymes in SWS correlates with the time elapsed from the AD diagnosis.
4. Reduction in stimulated saliva secretion in AD patients reflects secretory failure of the parotid glands. The function of the submandibular salivary glands responsible for unstimulated saliva secretion is completely impaired in the course of the disease.
5. Salivary glands dysfunction is reflected in the poorer hydration of the vermilion zone, buccal and tongue mucosa.
6. Secretory failure of the parotid glands may be associated with declined cholinergic conductivity; however, it could also depend on oxidative modification of proteins.

## Data Availability

The datasets generated for this study are available on request to the corresponding author.
